# Supplementary material for: Structures of the CcmABCD heme release complex at multiple states
Source: Nat Commun. 2022 Oct 28;13:6422. doi: 10.1038/s41467-022-34136-5 (PMC9616876; doi:10.1038/s41467-022-34136-5)

Supplementary Information

Structures of the CcmABCD Heme Release Complex at Multiple States

Supplementary Fig. 1

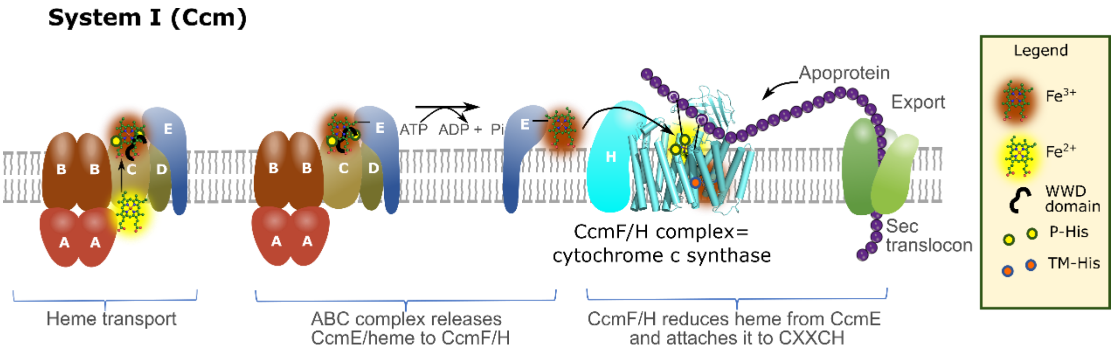

Supplementary Fig. 1 | Cytochrome *c* maturation (Ccm) system I.

The first step employs CcmABCD such that heme binds the external, periplasmic WWD/P-His domain in CcmC. (As described in the Discussion, it is unknown how heme traffics to the WWD/P-His domain in CcmC, here it is shown as passing from the cytoplasmic side of the membrane through CcmC to the CcmC WWD binding domain). CcmE then forms a covalent attachment signified by a solid line to heme (Fe^{+3}). Upon ATP hydrolysis by CcmA, holoCcmE is released from CcmABCDE: the present study describes the structural basis for this release mediated by the CcmABCD ABC transporter complex. Released holoCcmE shuttles heme to the CcmFH complex. In the WWD/P-His site of CcmF, heme is reduced from Fe^{3+} to Fe^{2+}, a heme reductase reaction mediated by a stable *b*-heme at the TM-site. P-heme (yellow) in CcmF is positioned at the CcmFH active site to bind apo cyt *c* (CXXCH), which has emerged through the Sec translocon. Upon covalent attachment of heme to cyt *c*, heme is released and cyt *c* folds.

Supplementary Fig. 2

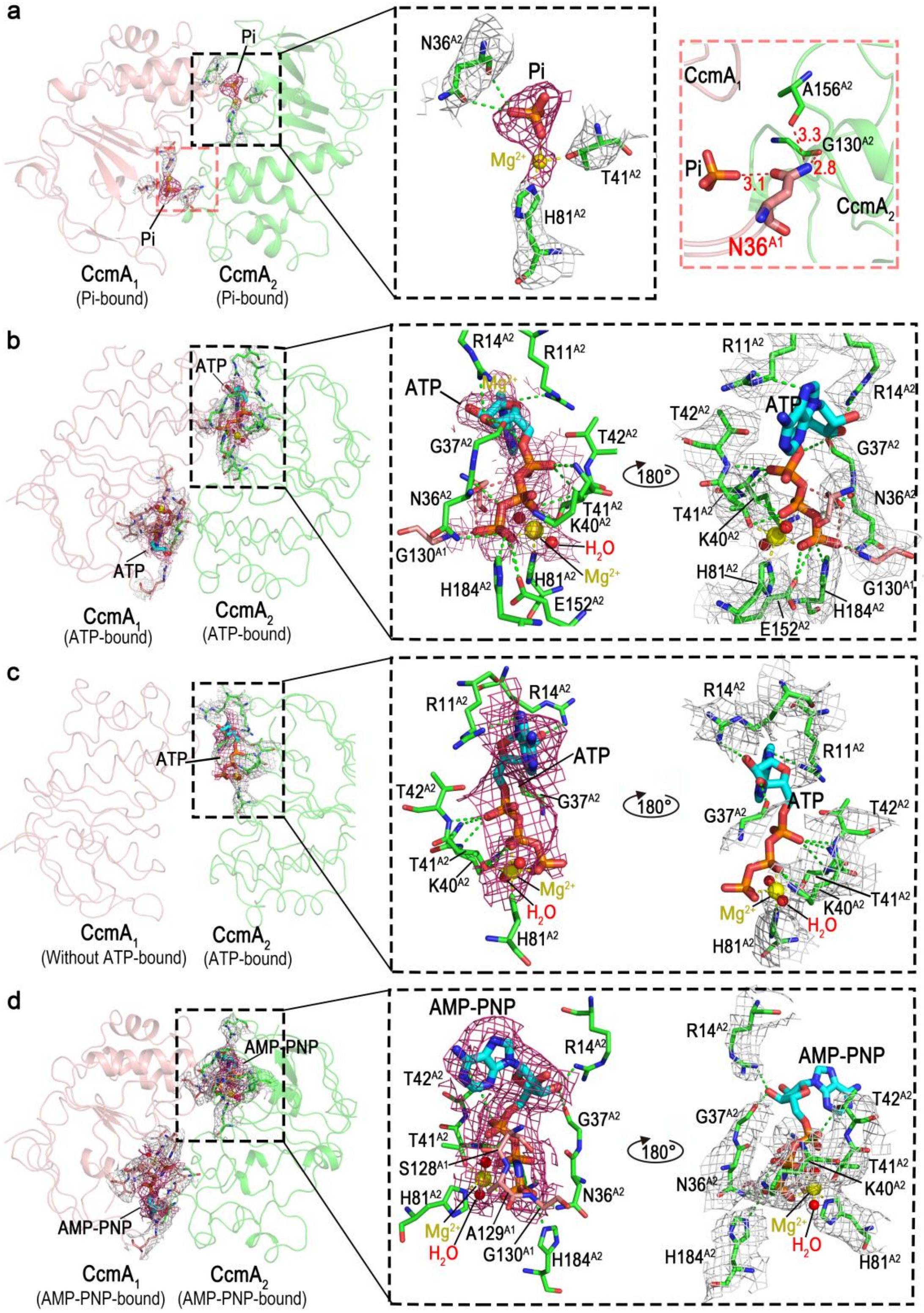

Supplementary Fig. 2 | ATP binding sites.

The cryoEM density of bound nucleotides, "putative" inorganic phosphates, Mg^{2+}, and waters is shown as warmpink mesh, and the density of key residues closely interacting

with the bound ligands is shown as grey mesh. **a**, The bound “putative” inorganic phosphates and key residues in the ATP binding sites of the apoCcmABCD closed NBD state (left zoomed in panel). $N36^{A}$ is located at the interface of two CcmAs, and H-bonded with the phosphate, and A156, G130 in other CcmA (right zoomed in panel). **b,** The bound ATPs and key residues in the ATP binding sites of the closed NBD state. **c**, The bound single ATP and key residues of the ATP binding site that is close to CcmC in the semi-open NBD state. **d**, The bound AMP-PNPs and key residues in the ATP binding sites of the closed NBD state.

Supplementary Fig. 3

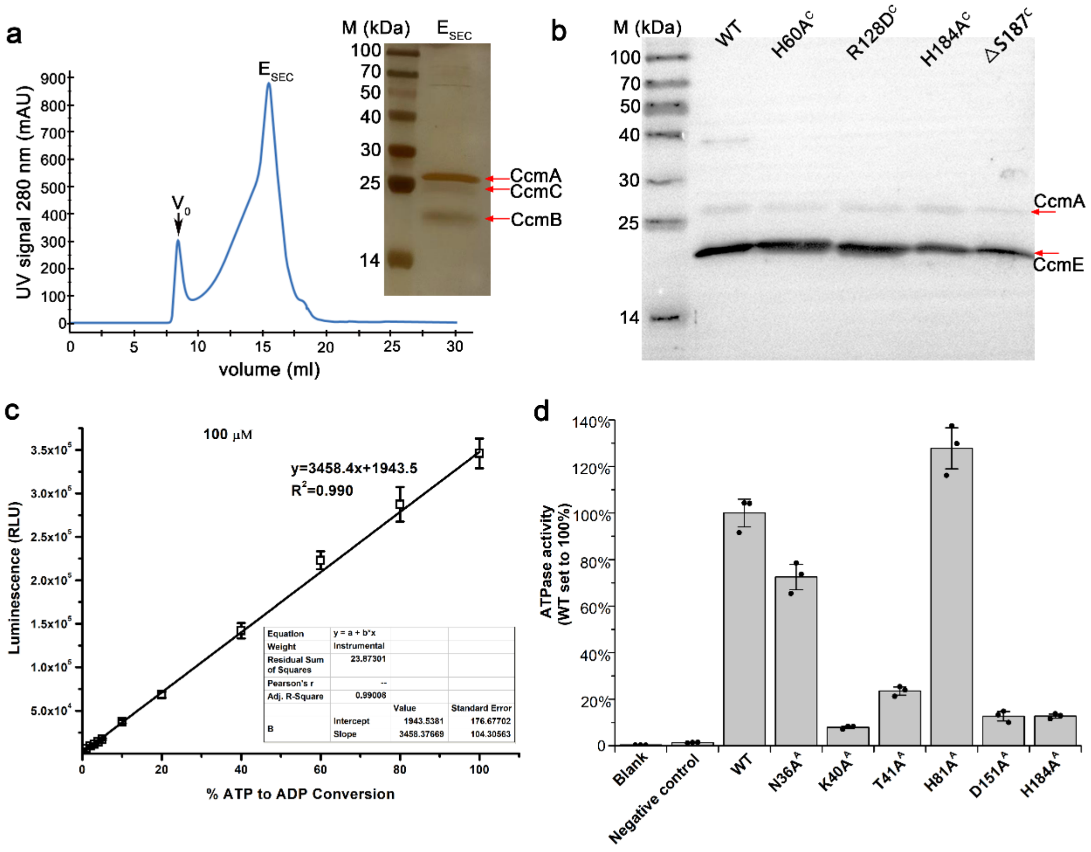

Supplementary Fig. 3 | Purification and ATPase activity assay of the WT and mutants of CcmABCDE complex.

a, FPLC SEC chromatogram of WT CcmABCDE displayed a sharp peak (E_{SEC}) at 15 ml on a Superose 6 10/300 GL column (V_0: void volume of the column). Inset: Silver-stained SDS-PAGE gel shows three protein bands between 14 and 30 kDa marker protein bands (M). The dark band represents the hydrophilic CcmA (23 kDa). The hydrophobic CcmB (23.6 kDa) and CcmC (27.9 kDa) show anomalous SDS-PAGE migration and different color of bands due to their hydrophobicity. There is no other band of hydrophilic protein feature, suggesting the absence of CcmE (17.5 kDa) in the gel. The experiment was repeated three times independently with similar results. **b**, Western blotting assay (anti-His-tag) of cell lysate sample of WT and mutants of CcmABCDE with His-tag on both CcmA and CcmE. The experiment was repeated

three times independently with similar results. The result show that the expression level of CcmABCDE is not significantly affected by mutations. **c**, Sensitivity and linearity of the ADP-Glo™ Kinase Assay. ATP-to-ADP conversion curves were prepared at the indicated ATP+ADP concentrations in 16 μl of reaction buffer in a solid white 384-well plate. There is a linear relationship between the luminescent signal and the amount of ADP in the reaction buffer at all ATP+ADP concentration series tested. Data are presented as mean ± s.d., n = 3, biologically independent experiments. Source data are provided in the Source Data file. **d**, ATPase activity assay for FPLC SEC purified WT and CcmA mutants. Data are presented as mean ± s.d., n = 3, biologically independent experiments. Source data are provided in the Source Data file. All of the ATP binding site mutants, except $N36A^{A}$ and $H81A^{A}$, show low ATPase activities.

Supplementary Fig. 4

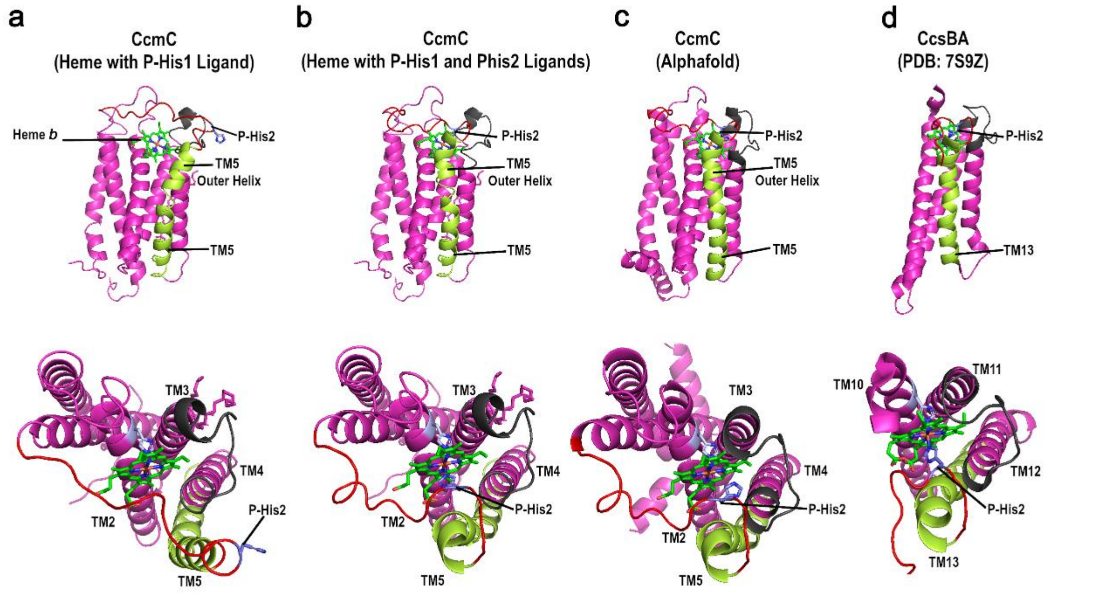

Supplementary Fig. 4 | Selected views of CcmC and CcsBA displaying P-His2 loop. **a**, Side view and top down view of CcmC structure with heme liganded by only the P-His1, TM5 is shown in yellow with its outer helix labelled and P-His2 loop shown in red. **b**, Side view and top down view of CcmC structure with heme liganded by both P-His1 and P-His2, TM5 is shown in yellow with its outer helix labelled and P-His2 loop shown in red. **c**, Side view and top down view of CcmC Alphafold model with no heme, TM5 is shown in yellow with its outer helix shown and P-His2 loop labelled in red. **d**, Side view and top down view of CcsBA Cryo-EM structure (PDB: 7S9Z) with heme liganded by both the P-His1 and P-His2, TM3 (CcsBA equivalent to TM5) shown in yellow and P-His2 loop shown in red.

Supplementary Fig. 5

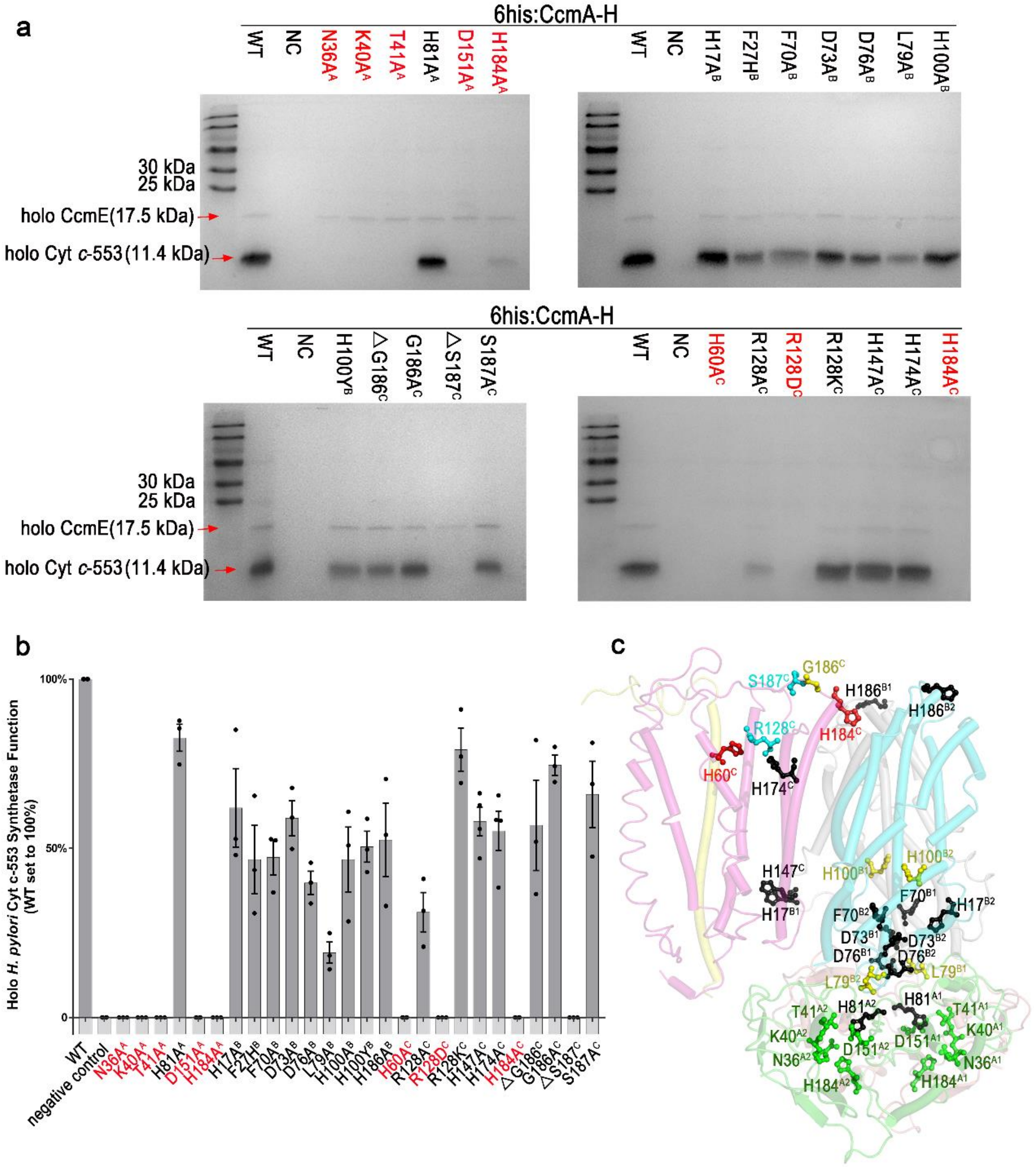

Supplementary Fig. 5 | Synthatase functions of mutants.

a, Representative heme staining gels of WT and mutants of CcmABCDEFGH. Each sample contains the same amount of cells of BL21-ΔCcm complemented with a constitutive plasmid incorporated with CcmA-H gene (WT or mutants) containing different point mutations and another inducible plasmid encoding the reporter gene *H. pylori* cytochrome *c*-553 (cyt *c*-553). Each sample was run on different gel for three times. Negative control only contains the reporter gene. **b**, Average synthetase

functions of WT and mutants of CcmABCDEFGH represented by bar chart. Data are presented as mean ± s.d. from of n = 3, where 'n' represents number of independent experiments. Source data are provided in the Source Data file. Mutants with no activities are highlighted with red. **c**, Mutation sites in this study. Residues in black: mutants of these sites have cyt *c* synthase activity higher than 50% of WT with normal holoCcmE production. Residues in red: mutants of these sites have no cyt *c* synthase activity and no holoCcmE production. Residues in green: mutants of these sites have no cyt *c* synthase activity but with holoCcmE production. Residues in yellow: mutants of these sites have cyt *c* synthase activity lower than 50% of WT, but have holoCcmE production. $R128^{C}$ and $S187^{C}$ are shown in Cyan. $R128A^{C}$: both cyt *c* and holoCcmE productions are significantly reduced; $R128D^{C}$: no cyt *c* synthase activity and no holoCcmE production; $R128K^{C}$: wild type phenotype; $S187A^{C}$: wild type phenotype; $\Delta S187^{C}$, no cyt *c* synthase activity and reduced holoCcmE production.

Supplementary Fig. 6

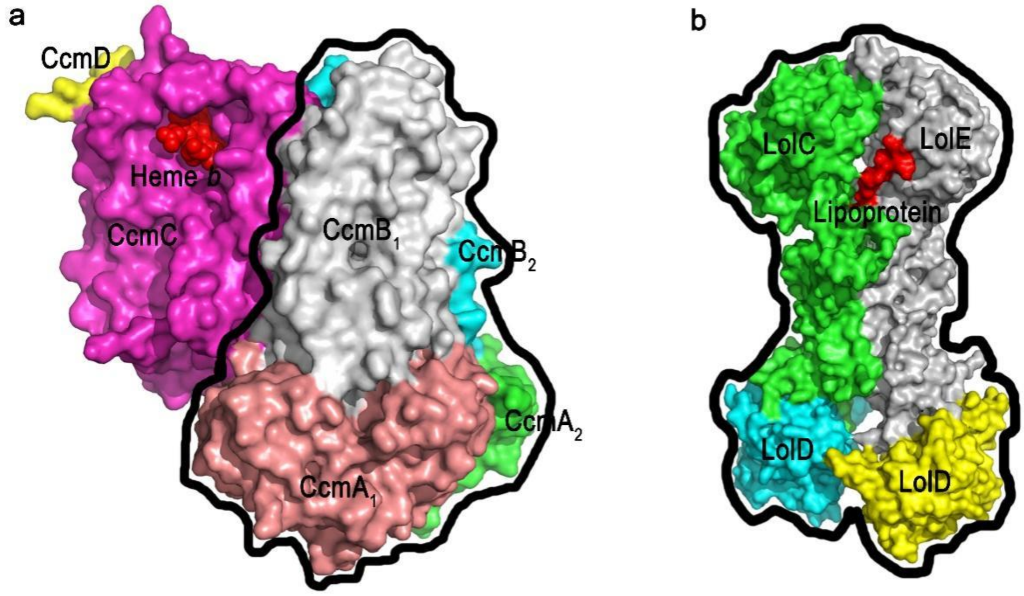

Supplementary Fig. 6 | CcmABCD ABC transporter release complex and LolCDE complex.

The ABC transporter modules are circled with black lines. **a**, CcmABCD release complex is composed of an ABC transporter structure $CcmA_2B_2$ and two attached subunits CcmC and CcmD. CcmC harbors the heme binding site and interacts two CcmBs with two coupling helices TM4 and TM5, respectively. **b**, The LolCDE protein complex (PDB: 7ARH). It consists of a heterodimer of transmembrane domains LolE and LolC and a homodimer of nucleotide-binding domains LolD.

Supplementary Fig. 7

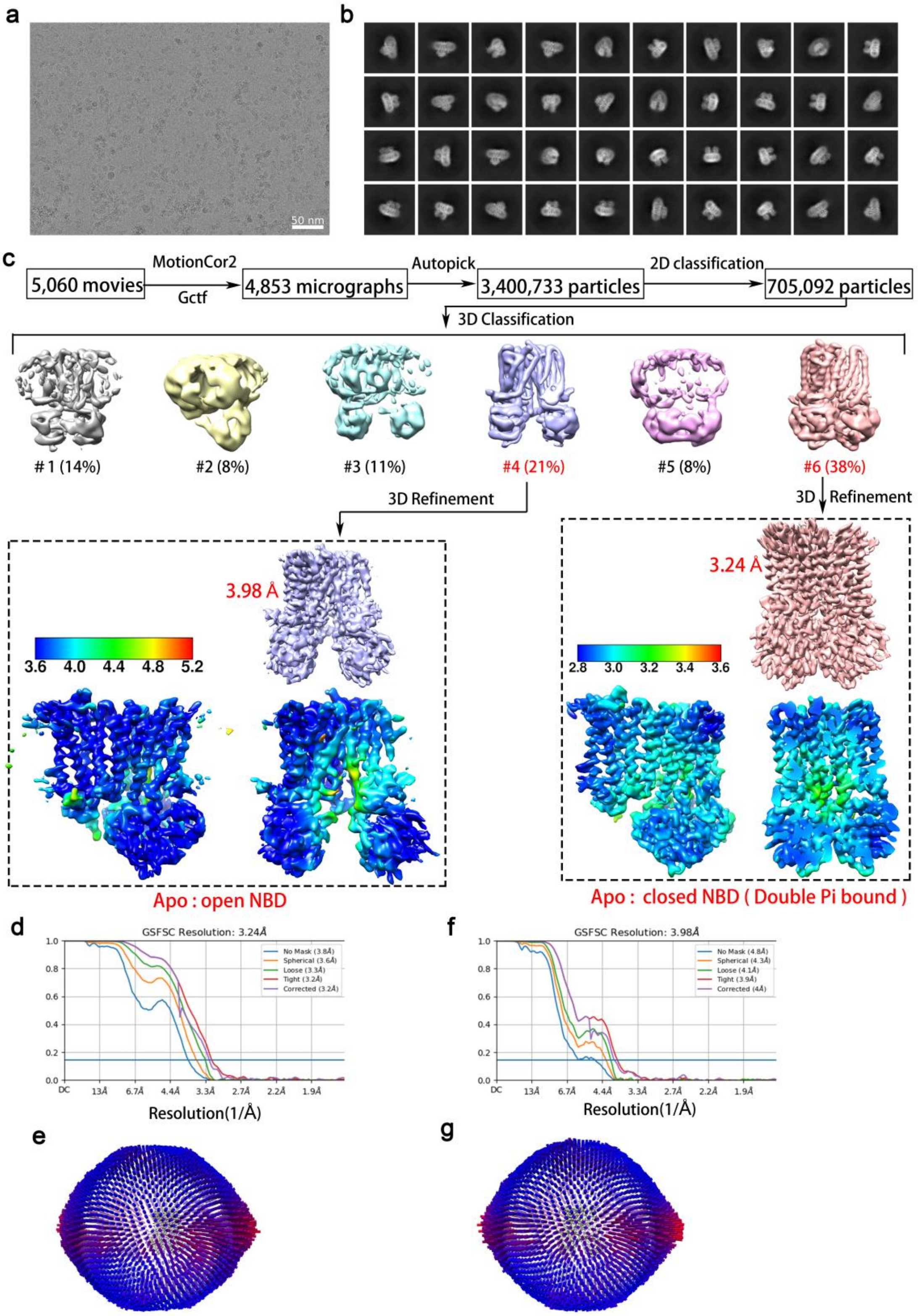

Supplementary Fig. 7 | Classification and refinement of the cryo-EM maps for *E. Coli* apo cytochrome *c* maturation complex CcmABCD.

The workflow, implemented in Relion 3.0.7 and cryoSPARC v2.15.0, was used to obtain the maps of open NBD conformation (ligand free) and closed NBD conformation (bound with two phosphates) at 3.98 Å and 3.24 Å resolution, respectively. **a**, Representative cryo-EM micrograph of apoCcmABCD embedded in vitreous ice. **b**, Forty representative 2D class averages of the final round of 2D classification, sorted in decreasing order by the number of particles assigned to each class. **c**, Flow chart for cryo-EM data processing and structure determination of apoCcmABCD in open NBD conformation (ligand free) and closed NBD conformation (bound with two phosphates). Two different views of the final 3D reconstructions were coloured according to their local resolution, as shown in the dashed boxes, with the clipping planes all in the upper left corner. **d**, Gold-standard Fourier shell correlation (FSC) curves calculated with different masks in cryoSPARC. The resolutions were determined at FSC = 0.143 (horizontal blue line). The final corrected mask gave an overall resolution of 3.24 Å of apoCcmABCD in closed NBD conformation. **e**, Angular distribution plot for the final reconstruction of the apoCcmABCD in closed NBD conformation. **f**, as in **d**, but for apoCcmABCD in open NBD conformation. The final corrected mask gave an overall resolution of 3.98 Å. **g**, as in **e**, but for apoCcmABCD in open NBD conformation.

Supplementary Fig. 8

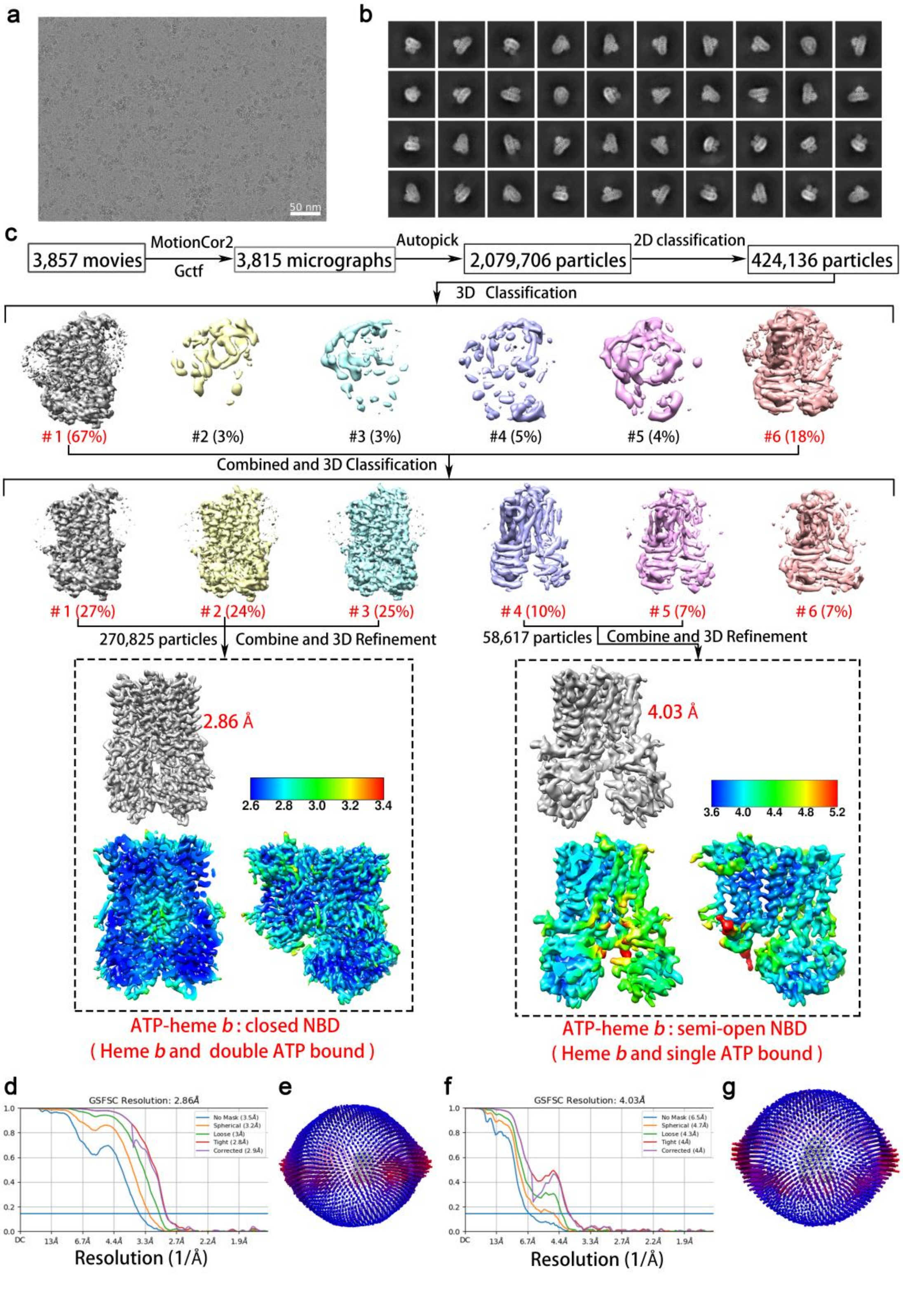

Supplementary Fig. 8 | Classification and refinement of the cryo-EM maps for *E. Coli* cytochrome *c* maturation complex CcmABCD bound with ATP and Heme *b*.

The workflow, implemented in Relion 3.0.7 and cryoSPARC v3.3.0, was used to obtain the maps of closed NBD conformation (bound with two ATPs) and semi-open NBD conformation (bound with one ATP) at 2.86 Å and 4.03 Å resolution, respectively. **a**, Representative cryo-EM micrograph of CcmABCD bound with ATP and Heme *b* embedded in vitreous ice. **b**, Forty representative 2D class averages of the final round of 2D classification, sorted in decreasing order by the number of particles assigned to each class. **c**, Flow chart for cryo-EM data processing and structure determination of CcmABCD in closed NBD conformation (bound with two ATPs) and semi-open NBD conformation (bound with one ATP). Two different views of the final 3D reconstructions were coloured according to their local resolution, as shown in the dashed boxes, with the clipping planes all in the upper right corner. **d**, Gold-standard Fourier shell correlation (FSC) curves calculated with different masks in cryoSPARC. The resolutions were determined at FSC = 0.143 (horizontal blue line). The final corrected mask gave an overall resolution of 2.86 Å of CcmABCD bound with two ATPs in closed NBD conformation. **e**, Angular distribution plot for the final reconstruction of CcmABCD bound with two ATPs in closed NBD conformation. **f**, as in **d**, but for CcmABCD bound with one ATP in semi-open NBD conformation. The final corrected mask gave an overall resolution of 4.03 Å. **g**, as in **e**, but for CcmABCD bound with one ATP in semi-open NBD conformation.

Supplementary Fig. 9

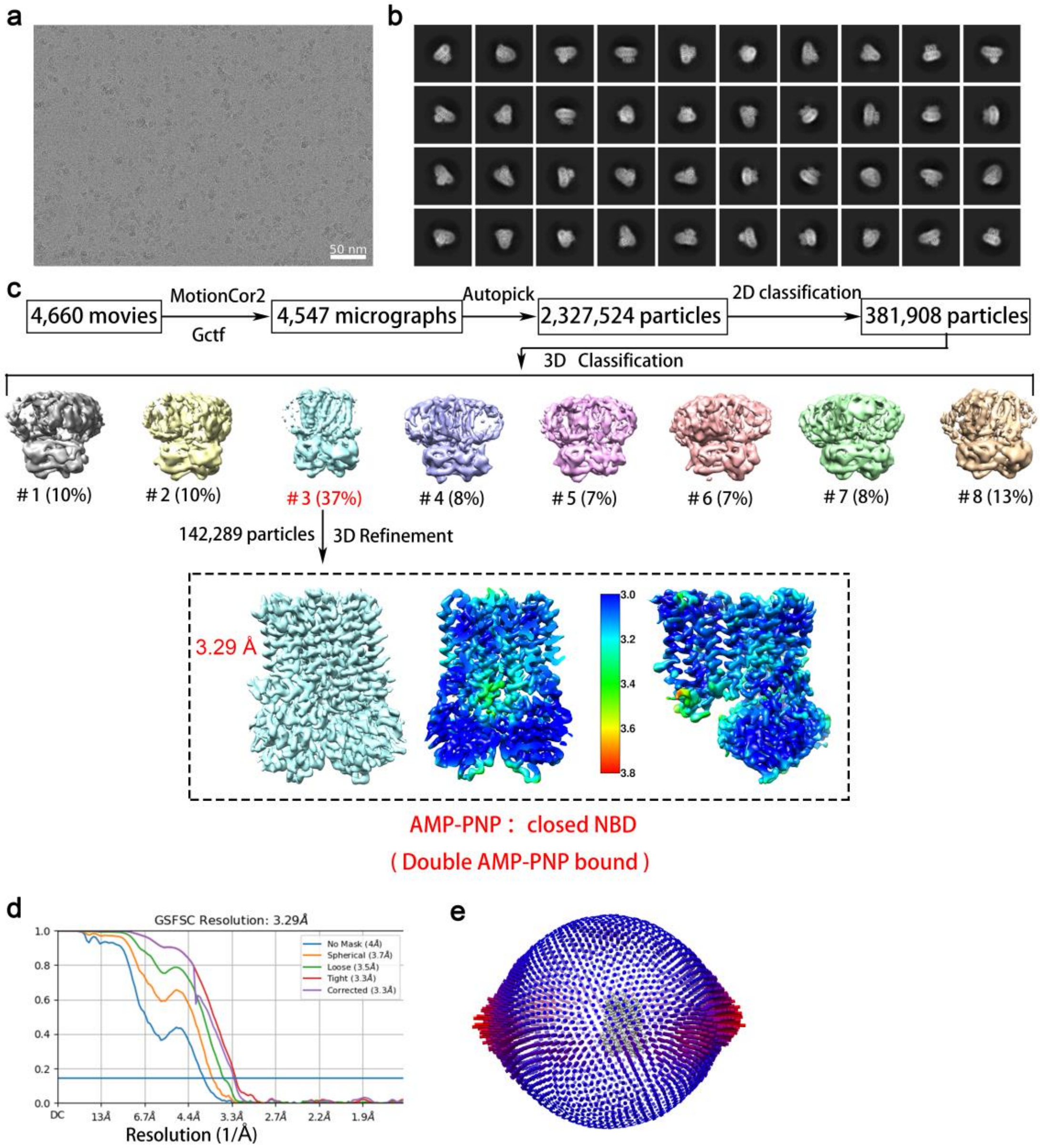

Supplementary Fig. 9 | Classification and refinement of the cryo-EM maps for *E. Coli* cytochrome *c* maturation complex CcmABCD bound with AMP-PNP.

The workflow, implemented in Relion 3.0.7 and cryoSPARC v2.15.0, was used to obtain the map of the closed NBD conformation bound with AMP-PNP at 3.29 Å. **a**, Representative cryo-EM micrograph of CcmABCD bound with AMP-PNP embedded in vitreous ice. **b**, Forty representative 2D class averages of the final round of 2D classification, sorted in decreasing order by the number of particles assigned to each

class. **c**, Flow chart for cryo-EM data processing and structure determination of CcmABCD bound with AMP-PNP. Two different views of the final 3D reconstruction were coloured according to their local resolution, as shown in the dashed box, with the clipping plane in the middle. **d**, Gold-standard Fourier shell correlation (FSC) curves calculated with different masks in cryoSPARC. The resolutions were determined at FSC = 0.143 (horizontal blue line). The final corrected mask gave an overall resolution of 3.29 Å of CcmABCD bound with AMP-PNP in closed NBD conformation. **e**, Angular distribution plot for the final reconstruction of CcmABCD bound with AMP-PNP in closed NBD conformation.

Supplementary Table 1. ATPase activities of the WT and CcmA mutants.

		ATPase activity (%) (mean ± s.d.*)
	WT	100% ± 5.9%
	Blank	0.3% ± 0.0%
	Negative control	1.3% ± 0.1%
CcmA mutants	N36A[A]	72.5% ± 5.4%
	K40A[A]	7.9% ± 0.6%
	T41A[A]	23.5% ± 1.7%
	H81A[A]	127.8% ± 8.8%
	D151A[A]	12.7% ± 2.0%
	H184A[A]	12.7% ± 0.9%

*Data are presented as mean ± s.d., n = 3, biologically independent experiments. Source data are provided in the Source Data file. All of the ATP binding site mutants, except N36A[A] and H81A[A], show low ATPase activities.

Supplementary Table 2. Primer list used in this study.

Gene	Primer Sequence (5'-3')
CcmABCDE	F: CATCATCATCACCATCATATGGGTATGCTTGAAGCCAGAGA<br>R: AGCTTGTCGACGGAGCTCTTATGATGCTGGGTCCTTATAAAC
CcmABCDEFGH	F: AACAGGGTTATTGCGTGGGT<br>R: CCGGCATAAACTAAGCGCAC
N36A[A]	F: CAAATCACCGGTAGCGCTGGCGCGGGGAA<br>R: CTTCCCCGCGCCAGCGCTACCGGTGATT
K40A[A]	F: AACGGCGCGGGGGCTACAACGCTTCTCC<br>R: CGGAGAAGCGTTGTAGCCCCCGCGCCGTTG
T41A[A]	F: GGCGCGGGGAAGGCTACGCTTCTCC<br>R: GGAGAAGCGTAGCCTTCCCCGCGCCG
H81A[A]	F: GTTATGGATAGGCGCCCAGCCGGGGATCAAAACCC<br>R: GGGTTTTGATCCCCGGCTGGGCGCCTATCCATAAC
D151A[A]	F: GTTATGGATCCTCGCTGAGCCTTTTACCGCGAT<br>R: ATCGCGGTAAAAGGCTCAGCGAGGATCCATAAC
H184A[A]	F: TGATTCTGACTACCGCTCAGCCGCTCAACGTTGC<br>R: GCAACGTTGAGCGGCTGAGCGGTAGTCAGAATCAC
H17A[B]	F: TAGCGTTTCGCGCCAGCGCCGAAATCGCCAAC<br>R: ATTTCGGCGCTGGCGCGAAACGCTACACGCAGCT
F70A[B]	F: CGCTGGAACGACTGGCGCGTGACGATTTGCAGGA<br>R: TCCTGCAAATCGTCACGCGCCAGTCGTTCCAGCG
D73A[B]	F: CTGTTCCGTGACGCGTTGCAGGACGGCAG<br>R: CTGCCGTCCTGCAACGCGTCACGGAACAG
D76A[B]	F: TGACGATTTGCAGGCGGGCAGTCTTGAACAATTGATG<br>R: CATCAATTGTTCAAGACTGCCCGCCTGCAAATCGTCAC
L79A[B]	F: GCAGGACGGCAGTGCGGAACAATTGATGTTGTTGCC<br>R: GGCAACAACATCAATTGTTCCGCACTGCCGTCCTGCAAAT
H100A[B]	F: GCGAAGGTGATGGCGGCTTGGATGGTAACCGG<br>R: CCGGTTACCATCCAAGCCGCCATCACCTTCGC
H186A[B]	F: GCTTCCATGGCTTTGCCCGTTGACGGGTATC<br>R: AACGGGCAAAGCCATGGAAGCCGCGTCCAT
H60A[C]	F: ATCTACCTGGCAGTGCCTGCGGCGATCTGG<br>R: ATCGCCGCAGGCACTGCCAGGTAGATAATGCGGTA
R128A[C]	F: GGGTATGGGATGCAGCACTGACTTCTGAACTGGTGC<br>R: GCACCAGTTCAGAAGTCAGTGCTGCATCCCATACCC
H184A[C]	F: TGGTGGAACACCCTGGCTCAGGGATCAACGC<br>R: GCGTTGATCCCTGAGCCAGGGTGTTCCACCAC
G186A[C]	F: ACACCCTGCATCAGGCTTCAACGCGGATGCA<br>R: CATCCGCGTTGAAGCCTGATGCAGGGTGT
ΔG186[C]	F: ACACCCTGCATCAGTCAACGCGGATGCA<br>R: TGCATCCGCGTTGACTGATGCAGGGTGT
S187A[C]	F: TGCATCAGGGAGCTACGCGGATGCAGCA<br>R: TGCTGCATCCGCGTAGCTCCCTGATGCA

ΔS187[C]	F: TGCATCAGGGAACGCGGATGCAGCAAAG<br>R: TTTGCTGCATCCGCGTTCCCTGATGCAG
H147A[C]	F: ATTGCCCTGTGGGCTGCCTTCGACGACCGC<br>R: GCGGTCGTCGAAGGCAGCCCACAGGGCAAT
H174A[C]	F: GCCGATTATTGCCTACTCCGTGGAGTGGTGGAACACC<br>R: GGTGTTCCACCACTCCACGGAGTAGGCAATAATCGGC

Supplementary Table 3. Cryo-EM data collection, refinement and validation statistics.

	ApoCcmABCD bound with inorganic phosphates (closed NBD)<br>(EMDB-31394)<br>(PDB 7F02)	Ligand-free apoCcmABCD (open NBD)<br>(EMDB-31956)<br>(PDB 7VFJ)	CcmABCD bound with AMP-PNP (closed NBD)<br>(EMDB-31395)<br>(PDB 7F03)	CcmABCD bound with heme *b* and double ATPs (closed NBD)<br>(EMDB-31396)<br>(PDB 7F04)	CcmABCD bound with heme *b* and single ATP (semi-open NBD)<br>(EMDB-31957)<br>(PDB 7VFP)
**Data collection and processing**					
Magnification	105,000×	105,000×	105,000×	105,000x	105,000x
Voltage (kV)	300	300	300	300	300
Electron exposure (e-/Å^2)	64.75	64.75	68.65	64.75	64.75
Defocus range (μm)	-1.2 to -2.0	-1.2 to -2.0	-1.2 to -2.0	-1.2 to -2.0	-1.2 to -2.0
Pixel size (Å)	0.832	0.832	0.832	0.832	0.832
Symmetry imposed	C1	C1	C1	C1	C1
Initial particle images (no.)	3400733	3400733	2327524	2079706	2079706
Final particle images (no.)	270353	147351	142289	270825	58617
Map resolution (Å)	3.24*	3.98*	3.29*	2.86*	4.03*
FSC threshold	0.143	0.143	0.143	0.143	0.143
**Refinement**					
Initial model used	-	PDB 7F02	PDB 7F02	PDB 7F02	PDB 7F02
Model resolution (Å)	-	3.24	3.24	3.24	3.24
FSC threshold	-	0.143	0.143	0.143	0.143
Map sharpening B factor (Å^2)	-	-80	-80	-80	-80
Model composition					
Nonhydrogen atoms	8837	8782	8893	8936	8859
Protein residues	1130	1130	1130	1130	1130
Ligands	5	0	5	6	5
B factors (Å^2)					
Protein	52.16	122.95	62.97	37.55	146.9
Ligand	55.91	-	65.01	39.5	152.72
R.M.S. deviations					
Bond lengths (Å)	0.003	0.004	0.003	0.004	0.004
Bond angles (°)	0.674	0.832	0.687	0.698	0.787
**Validation**					
MolProbity score	1.97	2.1	1.86	1.94	2.16
Clashscore	8.38	13.41	9.62	9.58	17.88
Poor rotamers (%)	0	0	0.11	0	0
Ramachandran plot					
Favored (%)	91.23	92.67	94.81	93.38	93.83
Allowed (%)	8.59	7.07	5.19	6.44	6.08
Disallowed (%)	0.18	0.27	0	0.18	0.09

*Determined with cryoSPARC.

Uncropped scans of all blots and gels presented in Supplementary Figures.

Supplementary Fig. 3a

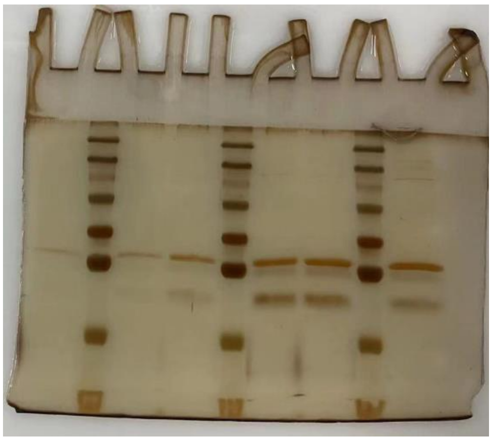

Supplementary Fig. 3b

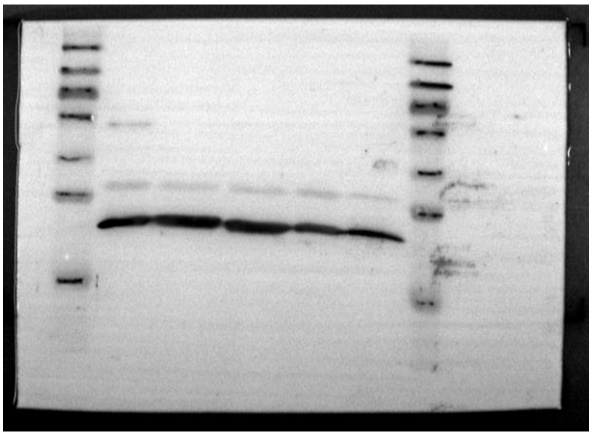

Supplementary Fig. 5a

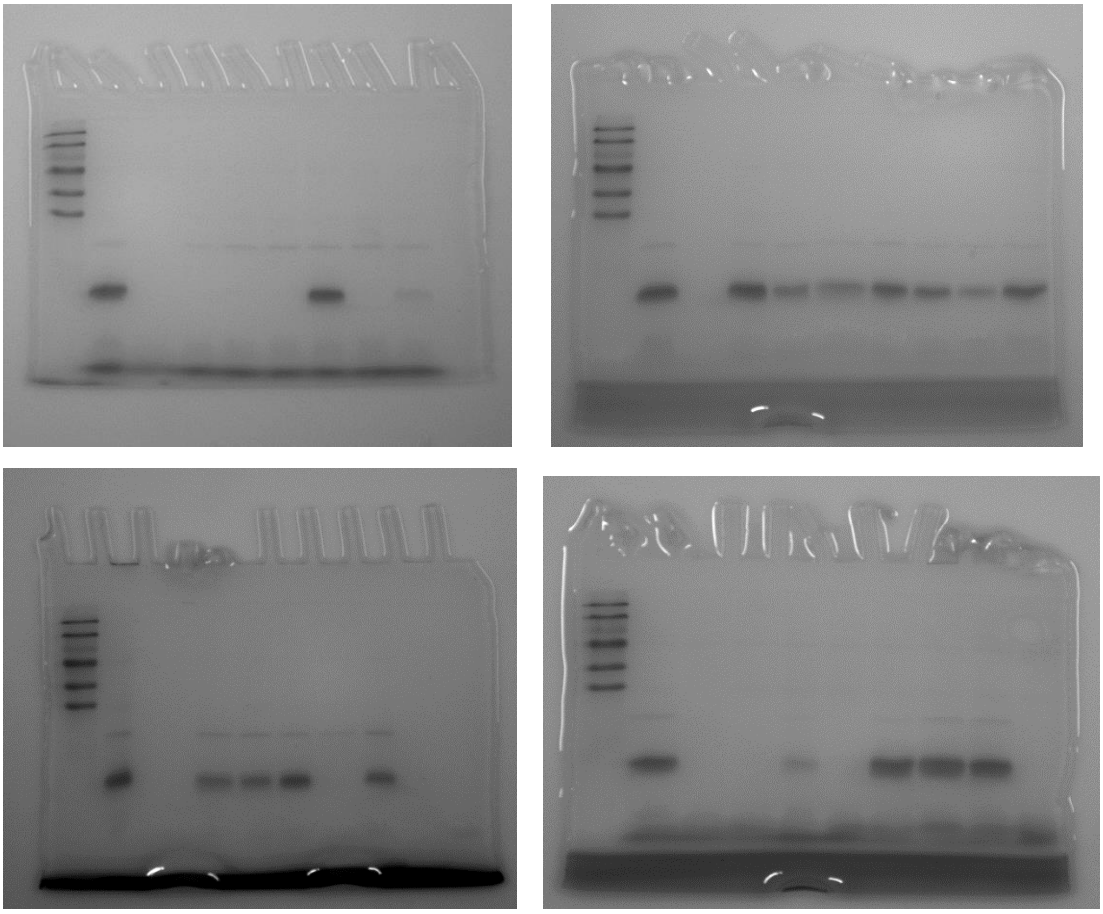

Supplement: Supplementary file 1 — Supplementary Information [file 41467_2022_34136_MOESM1_ESM.pdf]
