# Supplementary material for: Structures of the CcmABCD heme release complex at multiple states
Source: Nat Commun. 2022 Oct 28;13:6422. doi: 10.1038/s41467-022-34136-5 (PMC9616876; doi:10.1038/s41467-022-34136-5)
Supplement: Supplementary file 3 — Description of Additional Supplementary Files [file 41467_2022_34136_MOESM3_ESM.pdf]

File name: Supplementary Movie 1

Description: CcmABCD cycle of action showing the ATPase-based conformational changes that release heme (as holoCcmE).

The Movie begins with an enlargement of the CcmC active site and heme liganded by both P-His1 (red) and P-His2 (green), as well as the CcmE HDENY acceptor peptide (residues 130-134). His130^{E} (blue) is covalently linked to the heme 2-vinyl and Tyr134^{E} (orange). Tyr134^{E} will ultimately replace CcmC P-His2 upon conformational change catalyzed by the CcmA ATPase activity, thus moving from the closed to open NBD states. ATP is hydrolyzed into ADP and Pi. ADP and Pi are released as CcmABCD shifts to its open NBD conformation. Along with this nucleotide release, P-His2 (green) and CcmC outer TM5 shift away from heme iron to interact with CcmB1 (grey). Tyr134^{E} then moves to ligand heme allowing for the release of the heme (as holoCcmE). For simplification, only the CcmE acceptor sequence HDENY is shown, but the entire CcmE heme chaperone is bound and then release.
