# Supplementary material for: Structures of the CcmABCD heme release complex at multiple states
Source: Nat Commun. 2022 Oct 28;13:6422. doi: 10.1038/s41467-022-34136-5 (PMC9616876; doi:10.1038/s41467-022-34136-5)
Supplement: Supplementary file 5 — Reporting Summary [file 41467_2022_34136_MOESM5_ESM.pdf]

nature portfolio

Corresponding author(s): Robert G. Kranz, Kai Zhang, Jiapeng Zhu

Last updated by author(s): Oct 9, 2022

Reporting Summary

Nature Portfolio wishes to improve the reproducibility of the work that we publish. This form provides structure for consistency and transparency in reporting. For further information on Nature Portfolio policies, see our Editorial Policies and the Editorial Policy Checklist.

Statistics

For all statistical analyses, confirm that the following items are present in the figure legend, table legend, main text, or Methods section.

n/a	Confirmed	
☐	☒	The exact sample size (*n*) for each experimental group/condition, given as a discrete number and unit of measurement
☐	☒	A statement on whether measurements were taken from distinct samples or whether the same sample was measured repeatedly
☒	☐	The statistical test(s) used AND whether they are one- or two-sided<br>*Only common tests should be described solely by name; describe more complex techniques in the Methods section.*
☒	☐	A description of all covariates tested
☒	☐	A description of any assumptions or corrections, such as tests of normality and adjustment for multiple comparisons
☐	☒	A full description of the statistical parameters including central tendency (e.g. means) or other basic estimates (e.g. regression coefficient) AND variation (e.g. standard deviation) or associated estimates of uncertainty (e.g. confidence intervals)
☒	☐	For null hypothesis testing, the test statistic (e.g. *F*, *t*, *r*) with confidence intervals, effect sizes, degrees of freedom and *P* value noted<br>*Give P values as exact values whenever suitable.*
☒	☐	For Bayesian analysis, information on the choice of priors and Markov chain Monte Carlo settings
☒	☐	For hierarchical and complex designs, identification of the appropriate level for tests and full reporting of outcomes
☐	☒	Estimates of effect sizes (e.g. Cohen's *d*, Pearson's *r*), indicating how they were calculated

Our web collection on statistics for biologists contains articles on many of the points above.

Software and code

Policy information about availability of computer code

Data collection	Cryo-EM data was collected by SerialEM v3.7; ATPase assay data was collected by SpectraMax i3x
Data analysis	MotionCor2 v1.2.2; Gctf v1.18; Gautomatch v0.56; RELION v3.0.8; cryoSPARC v2.15.0; cryoSPARC v3.3.0; COOT v0.8.8; AlphaFold v2.0; ImageJ2 v2.3.0; Chimera v1.16; Phenix v1.14; ChimeraX v1.3; PyMOL v2.6.0; OriginPro 8.5

For manuscripts utilizing custom algorithms or software that are central to the research but not yet described in published literature, software must be made available to editors and reviewers. We strongly encourage code deposition in a community repository (e.g. GitHub). See the Nature Portfolio guidelines for submitting code & software for further information.

Data

Policy information about availability of data

All manuscripts must include a data availability statement. This statement should provide the following information, where applicable:

- Accession codes, unique identifiers, or web links for publicly available datasets
- A description of any restrictions on data availability
- For clinical datasets or third party data, please ensure that the statement adheres to our policy

Cryo-EM maps and atomic coordinates have been deposited in the Electron Microscopy Data Bank (EMDB) and the Protein Data Bank (PDB) under the accession codes EMD-31394 (https://www.ebi.ac.uk/emdb/EMD-31394) and 7F02 (https://www.rcsb.org/structure/7F02) (apoCcmABCD bound with inorganic phosphates in closed NBD state), EMD-31956 (https://www.ebi.ac.uk/emdb/EMD-31956) and 7VFJ (https://www.rcsb.org/structure/7VFJ) (ligand-free apoCcmABCD in open NBD

state), EMD-31395 (https://www.ebi.ac.uk/emdb/EMD-31395) and 7F03 (https://www.rcsb.org/structure/7F03) (CcmABCD bound with AMP-PNP in closed NBD state), EMD-31396 (https://www.ebi.ac.uk/emdb/EMD-31396) and 7F04 (https://www.rcsb.org/structure/7F04) (CcmABCD bound with heme b and double ATPs in closed NBD state), EMD-31957 (https://www.ebi.ac.uk/emdb/EMD-31957) and 7VFP (https://www.rcsb.org/structure/7VFP) (CcmABCD bound with heme b and single ATP in semi-open NBD state).

Human research participants

Policy information about studies involving human research participants and Sex and Gender in Research.

Reporting on sex and gender	no human research
Population characteristics	no human research
Recruitment	no human research
Ethics oversight	no human research

Note that full information on the approval of the study protocol must also be provided in the manuscript.

Field-specific reporting

Please select the one below that is the best fit for your research. If you are not sure, read the appropriate sections before making your selection.

☒ Life sciences ☐ Behavioural & social sciences ☐ Ecological, evolutionary & environmental sciences

For a reference copy of the document with all sections, see nature.com/documents/nr-reporting-summary-flat.pdf

Life sciences study design

All studies must disclose on these points even when the disclosure is negative.

Sample size	For cryoEM data, images were collected until the resolution and 3D reconstruction converges. For functional studies in this manuscript, we made various mutational studies of key residues in the active sites until we reached reliable conclusion, and each assay was performed in three biologically independent experiments, commonly exploited by researchers in this filed.
Data exclusions	CryoEM data processing involved removing poor-quality or damaged particles to achieve high resolution maps though pre-established standard data classification procedures.
Replication	Each experiment was repeated at least three times in independent experiments. Experimental findings were reproduced reliably.
Randomization	For the structural study, the data were collected and processed automatically and no manual randomization was needed. For functional studies in this manuscript, such as heme staining, western blotting and ATPase assay, there were no irrelevant variables that yielded biased statistical data, so randomization was not relevant.
Blinding	For all studies in this manuscript, such as 3D reconstruction, heme staining, western blotting and ATPase assay, there were no awareness of group assignment that caused biased results, so blinding was not relevant.

Reporting for specific materials, systems and methods

We require information from authors about some types of materials, experimental systems and methods used in many studies. Here, indicate whether each material, system or method listed is relevant to your study. If you are not sure if a list item applies to your research, read the appropriate section before selecting a response.

Materials & experimental systems

n/a	Involved in the study	
☐	☒	Antibodies
☒	☐	Eukaryotic cell lines
☒	☐	Palaeontology and archaeology
☒	☐	Animals and other organisms
☒	☐	Clinical data
☒	☐	Dual use research of concern

Methods

n/a	Involved in the study	
☒	☐	ChIP-seq
☒	☐	Flow cytometry
☒	☐	MRI-based neuroimaging

Antibodies

Antibodies used	His-Tag Polyclonal Antibody (Immunoway, Cat# YM3204, dilution: 1:5000); HRP Conjugated AffiniPure Goat Anti-Rabbit IgG (H+L) secondary antibody (Boster Biological Technology, Cat# BA1054, dilution: 1:5000)
Validation	His-Tag Polyclonal Antibody, Cat# YM3204, http://www.immunoway.com/Home/22/YM3204;<br>HRP Conjugated AffiniPure Goat Anti-Rabbit IgG (H+L) secondary antibody, Cat# BA1054, https://www.bosterbio.com/hrp-conjugated-goat-anti-rabbit-igg-secondary-antibody-ba1054-boster.html
